# Hand-washing at critical times and associated factors among mothers of children under-five in Ethiopia: a systematic review and meta-analysis

**DOI:** 10.1186/s13052-024-01801-y

**Published:** 2024-11-06

**Authors:** Gizaw Sisay, Negasa Eshete, Kassa Genetu

**Affiliations:** 1 School of Public Health, College of Medicine and Health Science, Dilla University, Dilla, Ethiopia; 2 Department of Environmental Health, College of Medicine and Health Science, Dilla University, Dilla, Ethiopia; 3 Department of Midwifery, College of Medicine and Health Science, Dilla University, Dilla, Ethiopia

**Keywords:** Hand-washing practice, Pooled proportion, Mothers of children under-five, Ethiopia

## Abstract

Good hand-washing practice is the most important preventive measure to reduce the spread of communicable disease to under-five children. Prior studies on good handwashing practice at critical times among mothers of children under-five age in Ethiopia were extremely varied**.** Therefore, this meta-analysis aimed to assess the overall proportion of good handwashing practice at critical times and identify the associated factors in Ethiopia. A comprehensive search of relevant studies was performed using distinct databases. Data were extracted using Microsoft Excel spreadsheet, and exported to Stata-MPv-17 for analysis. A random-effect model was used to estimate the overall proportion of good hand-washing practice at critical times. A random or fixed effects model was used to compute the pooled AORs with their 95% Cis, which were presented on a forest plot. ${I}^{2}$ test statistics was used for assessing heterogeneity among the included studies. The funnel plot and Egger's test were used to assess the publication bias. Ten studies were included in this systematic review and meta-analysis. The overall pooled proportion of good hand-washing practice at critical times among mothers of children under-five age in Ethiopia was 43.18% (95% CI = 31.05, 55.31). Insufficient water (AOR = 0.33, 95% CI: 0.13, 0.52), good knowledge (AOR = 1.35, 95% CI = 1.09, 1.61), desirable attitude (AOR = 4.34, 95% CI = 1.84, 6.84) and higher educational level (AOR = 0.17, 95% CI = 0.08, 0.25) were significantly associated with good practice of hand-washing at critical times. The national level of good practice of hand-washing at critical times was low. Hence, to promote good practice of hand-washing, it is essential to increase the accessibility of water and provide hygiene health education and training programs to improve knowledge and desirable attitudes of mothers.

## Main Text

One of the most crucial preventive measures against the spread of infectious diseases is proper hand-washing practice at critical times, a process of hand cleansing that dramatically lowers bacteria in the hands.

This study provides the first synthesis of evidence on proper hand -washing practice at critical times among mothers of children age below five years old in Ethiopian and reports estimates of the prevalence of proper hand -washing practice at critical times and the associated factors. This study highlights a huge gap in research on proper hand -washing practice at critical times among mothers of children age below five years old in Ethiopian.

This study highlights the need for improving maternal literacy, the availability of water sources in the backyard, and the positive attitude of mothers are needed to maintain and enhance proper hand-washing practice at critical times.

## Introduction

Proper hand-washing practice at critical times is are components of personal hygiene that helps prevent diseases, save lives, promote good health, and contribute to a country’s social and economic growth [1, 2].

According to the Center for Disease Control, the critical times for proper hand washing are: after toilet, after changing diapers, after caring for a sick person, after handling raw meat, fish or poultry, and after handling waste [3]. Public health guidelines recommend handwashing with water and soap before cooking food, before feeding a child, before eating food, after defecation, and after cleaning a child who has defecated [4, 5].

Diarrhoea and pneumonia are the leading causes of death among children below 5 years old due to inadequate sanitation and hygiene, resulting in approximately 1.8 million deaths worldwide annually [6]. In Ethiopia, more than 70,000 children die each year due to diarrheal diseases [7].

Worldwide, the mortality rates of under-five aged children remain high. In 2013, 6.3 million children were documented to have died before the age of five. Of these, 51.8% (3.26 million) died due to infectious causes. If current trends continue, 4.4 million children under the age of five are expected to die by 2030. Furthermore, Sub-Sahara African countries will have 33% of births and 60% of deaths [8].

Mothers and other children caretakers engage in various activities such as washing their children’s bottoms, maintaining a clean home and yard, interacting with domestic animals, and visiting restrooms where their hands come into contact with microorganisms. The adherence of mothers to proper hand-washing practice can reduce the incidence of pneumonia and diarrhea among children under-five years old [9].

A recent study has shown that proper hand washing at critical times can significantly reduce bacterial infections and new confirmed cases of COVID-19 [10].

The Sustainable Development Goals aim to ensure healthy lives for all children and decrease the number of deaths among under-five children by 10 million between 2017 and 2030. This can be achieved by improving good hand-washing practice at critical times, as the hands play a central part in our daily activities. The uses of contaminated hands during cooking and eating increases the transmission of microbes that can cause illness [11].

According to the 2019 joint WHO-UNICEF report, hand hygiene is often neglected at critical times due to the lack of access to hand washing facilities in key areas [12]. Recent report showed that an estimated 40% of the world’s population lack basic handwashing facilities at home. Over 50% of the population in sub-Saharan Africa and Oceania do not have access to a facilities equipped with water and soap [12].

Proper hand-washing is rarely practiced in low-income countries such as Ethiopia. Previous research findings suggested that proper hand-washing at critical times such as after defecation or cleaning an infant’s perineum is not a common practice[13].

The proportion of proper hand-washing practice at critical times among mothers of children under-five age was different across countries around the world. It was 76% India [9], 41.6% in Nigeria [14], 21% in Indonesia [15], 67% in Bangladesh [16], in Uganda ranges from 28.5% to 71.4% [17] and 65% in Kenya [18].

Evidence showed that different factors affect the practice of good handwashing practice at critical times among mothers of children under-five years of age. Educational status, knowledge, attitude, availability of water and family size greater than five were significantly associated with good handwashing practice at critical times [19–22].

A research studies conducted in Ethiopia examining good handwashing practice at critical times among mothers of children under-five reported inconsistent results, ranged from 19.8% [23] to 74.4% [24]. Hence, this review and meta-analysis aimed to estimate the pooled proportion of good hand-washing practice at critical times and its associated factors in Ethiopia. The result of this study will provide evidence for policymakers and for program evaluators.

### Methods

This systematic review and meta-analysis was performed according to the Preferred Reporting Items for Systematic Reviews and Meta-Analyses (PRISMA) 2020 guidelines [25].

### Search strategies

A comprehensive search of relevant studies published in English language was undertaken in various databases such as PubMed, DOJA, Embase, Science Direct, Cochrane Library, African Journals online, Google scholar, and Web of Science). Initially,studies were comprehensively searched using the full title (“The proportion and associated factors of hand-washing practice at critical times among mothers of children under-five in Ethiopia”) and keywords (“magnitude,” “proportion,” “hand-washing practice at critical times,” hand-cleaning”, “hand-hygiene”, “determinant factors,” “associated factors,” “predictors,” “in Ethiopia”). Boolean operators “OR” or “AND” were used in combination or independently to connect these keywords and to establish the search terms. Additionally, thereference lists of all included studies were to identify other missed publications.

### Eligibility criteria

To determine the inclusion and exclusion criteria for this systematic review and meta-analysis, population, intervention, comparison, and outcomes technique was employed, which involved the uses of condition, context, and population questions for prevalence studies [26].

### Inclusion criteria

- **Study area:** studies conducted in Ethiopia.
- **Population:** Low-risk mothers of children below 5 years old
- **Study setting:** Studies conducted in health care facilities or community-based settings.
- **Study design:** All observational studies (cross-sectional, cohort, and case–control studies that reported either the proportion or magnitudes and associated factors or predictors of handwashing practice at critical times.

### Exclusion criteria

Qualitative studies that didn’t provide quantitative evidence and those that used different operational definitions and measurements of good hand-washing practice at critical times were excluded from this review and meta-analysis.

### Data extraction

Data from the selected studies were separately extracted using a predesigned data extraction form in Microsoft Excel version 2016. The spreadsheet included the first author’s name, year of the data collection conducted, year of publication, study design, study area, study setup, sample size, and the proportion good practice of hand-washing at critical times. Any discrepancies between the data extractors were resolved through discussion and re-evaluation of the studies.

### Quality assessment

The Joanna Briggs Institute (JBI) Critical appraisal checklist was used to evaluate the methodological quality of the included studies [27]. There are nine parameters in the tool. The two authors separately ranked the quality of the included studies.

Each item was evaluated as low, moderate or high risk of bias. A composite quality index was categorized and the risk of bias was ranked as low (0–2), moderate (3 or 4), or high (≥ 5).

### Outcomes

This study had two outcomes (proportion and associated factors of good hand-washing practice at critical times among mothers of children under-five in Ethiopia. Based on the operational definition of the included studies; good hand-washing practice at critical times was considered when the respondents who scored above the mean of the practice related questions.

### Data management and statistical analysis

Data were extracted using a Microsoft Excel spreadsheet and analysed using the Stata 17 statistical software. The Higgins ${I}^{2}$ statistic and Cochran’s Q-test were used to examine the presence of statistical heterogeneity among the included studies. A random-effects model using the DerSimonian-Laird method was used to estimate the pooled prevalence of proper handwashing practices at critical times. The adjusted odds ratios (AORs) and 95% confidence intervals (CIs) were extracted from the included studies. A random- or fixed-effects model was used to calculate the pooled AORs. Lastly, the pooled estimates for proper handwashing practices at critical times and the associated factors, as well as their corresponding 95% CIs, were presented in forest plots.

### Subgroup analyses and heterogeneity

To identify the potential sources of heterogeneity among the included studies, subgroup analyses were conducted based on the sampling method, geographic region, and year of the study.

### Publication bias

Publication bias was visually examined using funnel plots and statistical methods, including Egger’s and Begg’s tests.

### Sensitivity analysis

To assess the impact of individual studies on the overall prevalence of proper handwashing practices at critical times, a sensitivity analysis was performed using a random-effects model.

## Results

**Fig. 1:**
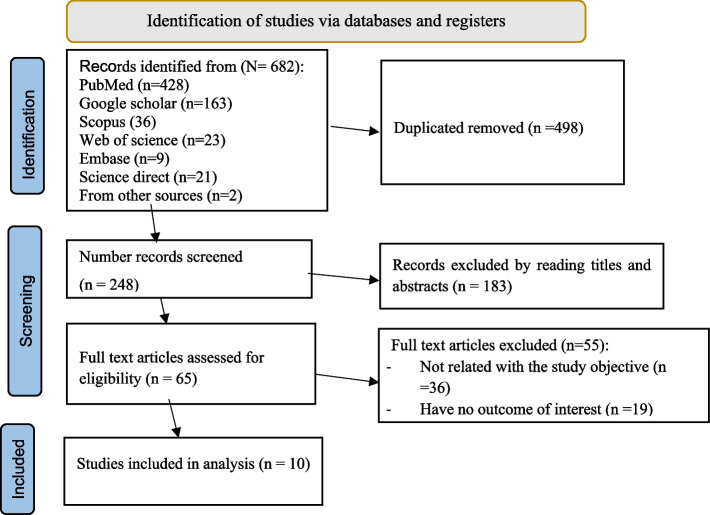
PRISMA 2020 flow diagram describing the selection of studies for systematic review and meta-analysis

Various databases were searched to identify relevant studies for this systematic review and meta-analysis, resulting in the retrieval of 682 published studies. Among these, 498 duplicate studies were excluded. The Endnote Reference Manager software was used to refine the selection. After screening the titles and abstracts, 65 articles were identified. Of these, 55 studies were excluded as their titles and abstracts did not meet the inclusion criteria. Finally, only ten studies that met the inclusion criteria were included in this meta-analysis (Fig. 1).

### Characteristics of included studies

This review and meta-analysis included ten articles [23, 24, 28–35] with a total of 4,531 participants. Data collection across these studies was conducted through interviews using a pretested, interviewer-administered questionnaire. The included articles were published from 2014 to 2022. Three studies were from the Amhara region [29, 34, 35]; two were from the Oromia region [30, 33]; three were from the Southern Nations, Nationalities, and Peoples’ Region (SNNPR) [28, 31, 32] and two were from the Addis Ababa City administration [23, 24]. All studies used a cross-sectional design and had a low risk of bias (Table 1).

**Table 1:** Presents a descriptive summary of the studies included in this systematic review and meta-analysis

Author, Publication Year	Study Year	Study Area	Region	Settings	Design	Sampling method	Sample size	Prevalence (%)	Risk of bias
Adane, M. et al., 2018 [23]	2014	Gulele	AA	CB	CS	M/stage	690	19.80	Low risk
Agaro, A. et al., 2022 [28]	2021	Dilla Zuria	SNNP	IB	CS	SyRS	422	44.90	Low risk
Dagne, H. et al., 2019 [29]	2018	Debark town	Amhara	CB	CS	SRS	402	52.20	Low risk
Demssie, A. et al., 2017 [30]	2015	Wondogenet	Oromia	CB	CS	SyRS	251	29.91	Low risk
Mekonen, T. et al., 2021 [31]	2020	Daworo zone	SNNPR	CB	CS	M/stage	520	21.50	Low risk
Meleko, A. 2018 [32]	2017	Bench Maji	SNNPR	CB	CS	SRS	422	34.60	Low risk
Negasa E. et al., 2022 [33]	2018	Jimma Zone	Oromia	CB	CS	M/stage	756	64.4	Low risk
Taddese AA. et al., 2020 [34]	2019	UoG, Hospital	Amhara	IB	CS	SRS	422	39.10	Low risk
Wana E.W. 2023 [24]	2019	Lafto Sub-City	AA	IB	CS	SRS	312	74.40	Low risk
Wolde M., et al., 2022 [35]	2020	Koladiba	Amhara	CB	CS	SyRS	334	51.20	Low risk

*CS* Cross-sectional study, *CB* community based, *IB* institutional based, *SRS* simple random sampling, *SyRS* systematic random sampling, *M/stage* multistage sampling, *AA* Addis Ababa, *SNNPR* Southern Nation Nationalities and Peoples of Ethiopia Region

### The pooled proportion of good handwashing practice at critical in Ethiopia

**Fig. 2:**
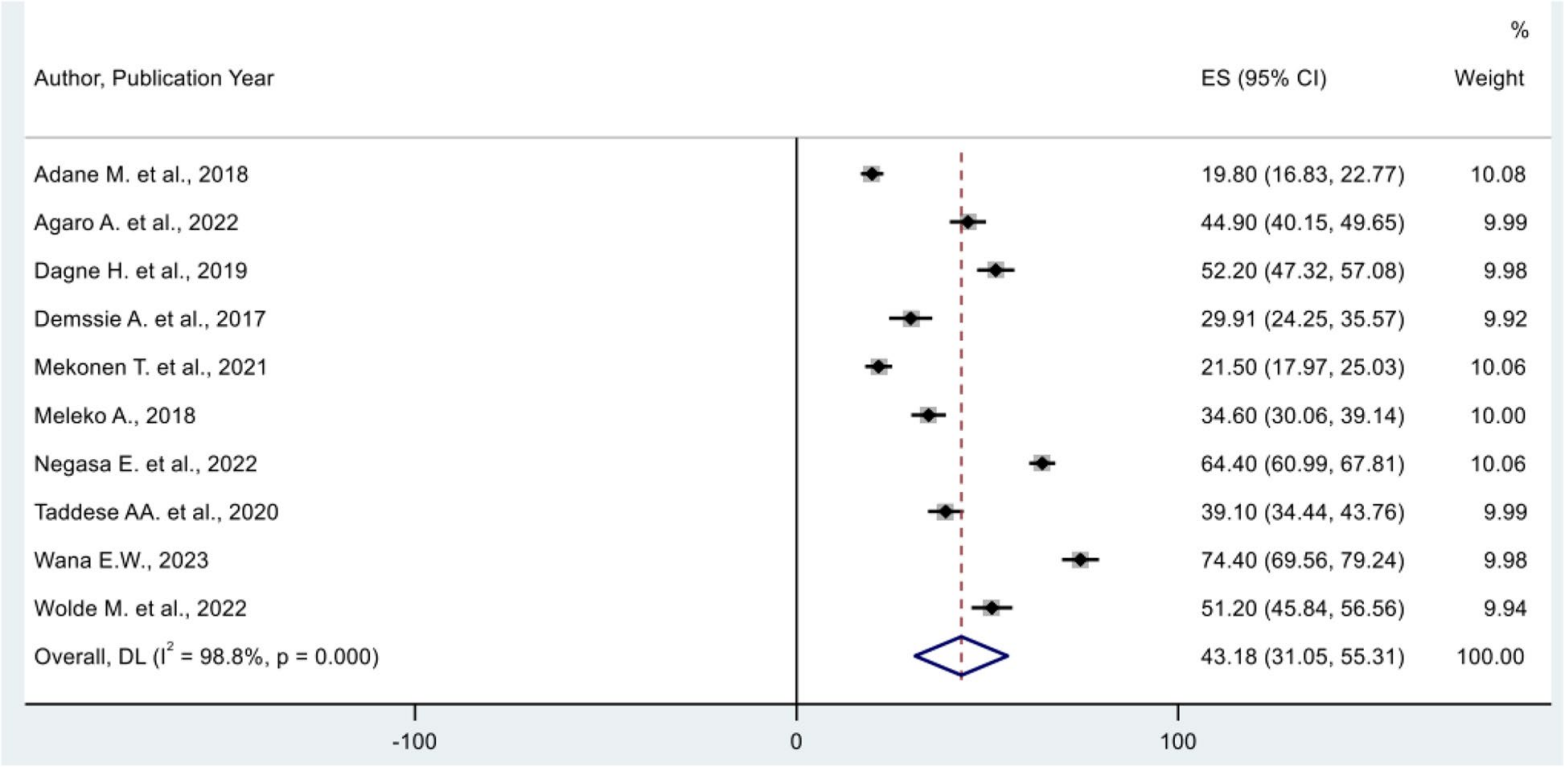
Forest plot showing the pooled proportion estimate of good hand-washing practice at critical times in Ethiopia

The overall pooled proportion of good hand-washing practice at critical times among mothers of children under-five in Ethiopia was 43.18% (95% CI = 31.05, 55.31) (Fig. 2). An ${I}^{2}$ test was conducted to examine the heterogeneity of the included studies, and revealed a high heterogeneity across all the included studies (${I}^{2}$=98.8, P-value = 0.00). Therefore, a random-effects model employing DerSimonian–Laird method was used to estimate the pooled proportion of good hand-washing practice at critical times. Among the included studies, the highest proportion of good hand-washing practice at critical times was 74.4 with 95%CI (69.56, 79.24) reported by Wana E.W. et al., 2023 [24], and the lowest proportion of good hand-washing practice at critical times was 19.80 with (95% CI: (16.83, 22.77%) reported by Adane, M. et al., 2018 [23].

### Sensitivity analysis

**Fig. 3:**
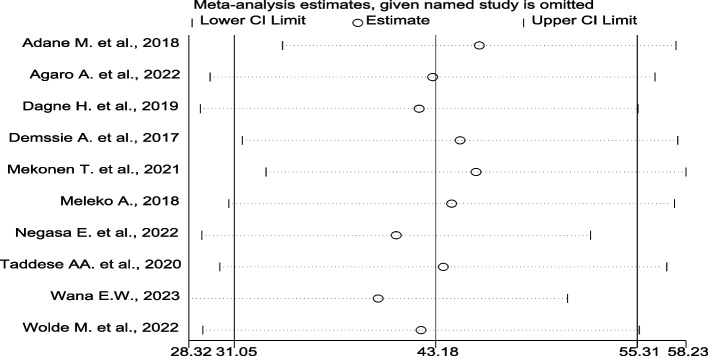
Sensitivity analysis of the level of handwashing at critical times removed at a time and 95% confidence limits

A leave-one-out sensitivity analysis using the random-effects model revealed that there is no single study that influenced the pooled estimate. After eliminating a single study from the analysis, the pooled proportion of good hand-washing practice at critical times was close to the actual effect size (Fig. 3).

### Publication Bias

**Fig. 4:**
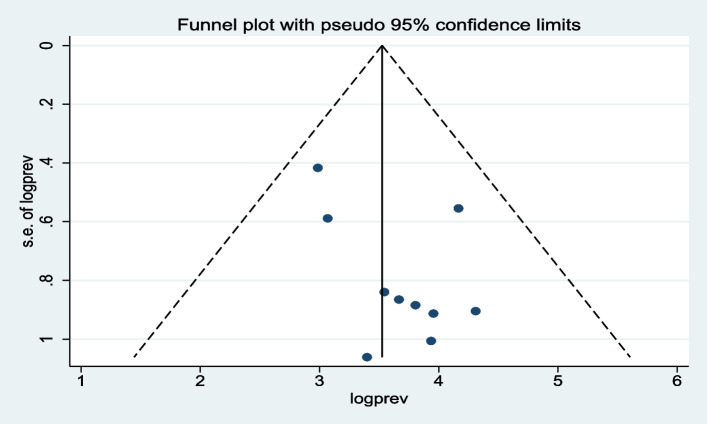
Funnel plot with 95% confidence limits of the pooled proportion of good hand-washing practice at critical times among mothers of children under-five in Ethiopia

To evaluate publication bias, a graphical funnel plot was used. Visual inspection of the plot revealed asymmetry, indicating the presence of publication bias (Fig. 4). Furthermore, Egger’s regression test was conducted to determine the statistical significant of observed. The results of Egger’s regression test indicated no significant small-study effects among the included studies (p = 0.277). Therefore, the observed publication bias on the funnel plot was not significant and likely attributable to chance.

### Handling heterogeneity

#### Subgroup analysis

To assess and balance the source of heterogeneity for the pooled proportion of good hand-washing practice at critical times, subgroup analysis were performed by geographical region, year of the study conducted and sampling techniques of the included studies. The overall pooled proportion of good hand-washing at critical times was almost the same in the three geographical regions (AA, Amhara and Oromia) of Ethiopia. However, studies conducted in SNNP region of Ethiopia had the lowest proportion of good hand-washing practice at critical times 33.06% (95% CI: 19.82, 47.37). Furthermore, we performed a subgroup analysis based on the year the studies were conducted. Thus, the pooled proportion of good hand-washing practice at critical times was higher for those studies conducted in 2019 and after (44.94%, 95% CI = 22.86, 6702) as compared to for those studies conducted before 2018 (42.02%, 95% CI = 25.99, 58.04) (Table 2).

**Table 2:** Sub-group analysis for the pooled proportion of good hand-washing practice at critical times in Ethiopia

Variables	Subgroup	No. of included studies	Proportion of hand-washing at critical times (95% CI)	Heterogeneity across the studies		Heterogeneity between group (p-value)
				${I}^{2}$(%)	P-value	
Region	AA	2	47.07(6.44, 99.8)	99.7	0.00	0.413
	Amhara	3	47.44(38.91, 55.98)	88.5	0.00	
	Oromia	2	47.23(13.43, 81.03)	99.0	0.00	
	SNNPR	3	33.06(19.82, 47.37)	96.8	0.00	
Year of study conducted	2014–2018	5	42.01(25.99, 58.04)	99.0	0.00	0.834
	2019–2022	5	44.94(22.86, 67.02)	98.7	0.00	
Sampling method	SRS	4	50.06(32.63, 67.50)	98.2	0.00	0.628
	SyRS	3	42.05(30.31, 53.79)	93.3	0.00	
	M/stage	3	35.23(6.97, 63.49)	99.5	0.00	

Lastly, a subgroup analysis was also conducted on the type of sampling method the included studies used. As a result, studies with simple random sampling had a highest proportion of good hand-washing practices at critical times (Table 2).

## Factors Associated with Good Hand-washing practice at Critical times

Seventeen variables were extracted from the included studies to identify factors associated with good hand washing practice at critical times. Finally, four variables (insufficient water, educational status, good knowledge and desirable attitude were significantly associated with good hand-washing practice at critical times.

**Fig. 5:**
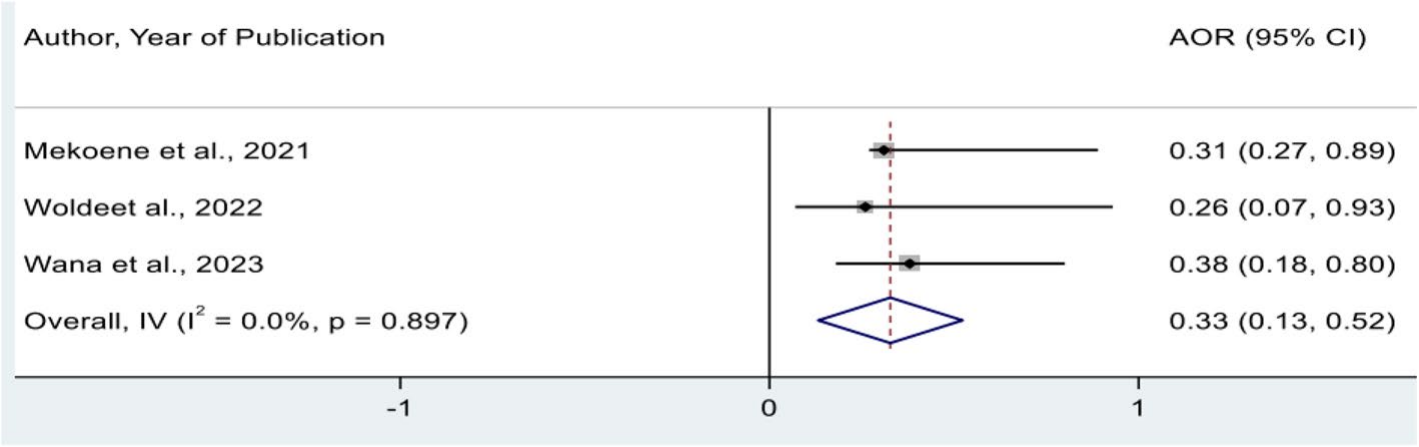
Forest plot showing the pooled adjusted odds ratio for the association between insufficient water and good hand-washing practice at critical times in Ethiopia

The effect of insufficient water for good hand-washing at critical times was assessed by using the findings of 3 studies [24, 31, 35]. In this meta-analysis, mothers of children under-five who had no sufficient water were 77% less likely to practice good hand-washing practice at critical times than those who had a sufficient water (POR = 0.33, 95% CI: 0.13, 0.52) (Fig. 5).

**Fig. 6:**
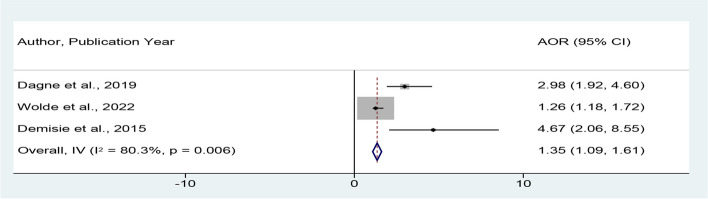
*Forest plot showing the* pooled adjusted odds ratio for the *association between knowledge and good hand-washing practice at critical times in Ethiopia*

The estimated pooled effect of good knowledge on good hand-washing at critical times among mothers of children under-five was assessed by using three studies [29, 30, 35]. In this study, knowledgeable mothers were 1.35 times more likely to have good hand-washing practice at critical times than those with poor knowledge about hand washing at critical times (POR = 1.35, 95% CI = 1.09, 1.61) (Fig. 6).

**Fig. 7:**
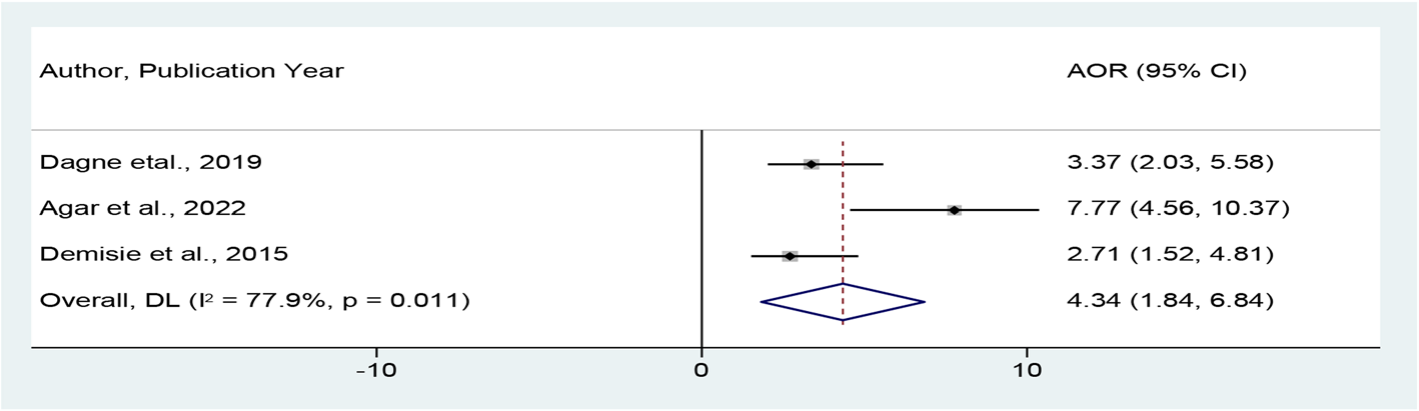
Forest plot showing the pooled adjusted odds ratio for the association between positive attitude and good handwashing at critical times in Ethiopia

Also, three studies [28–30] were used to assess the pooled odd ratio for the association between positive attitude and good handwashing at critical times. Accordingly, mothers of children under-five who had desirable attitude were 4.34 times more likely to practice proper hand-washing at critical times as compared to the counterparts (POR = 4.34, 95% CI = 1.84, 6.84) (Fig. 7).

**Fig. 8:**
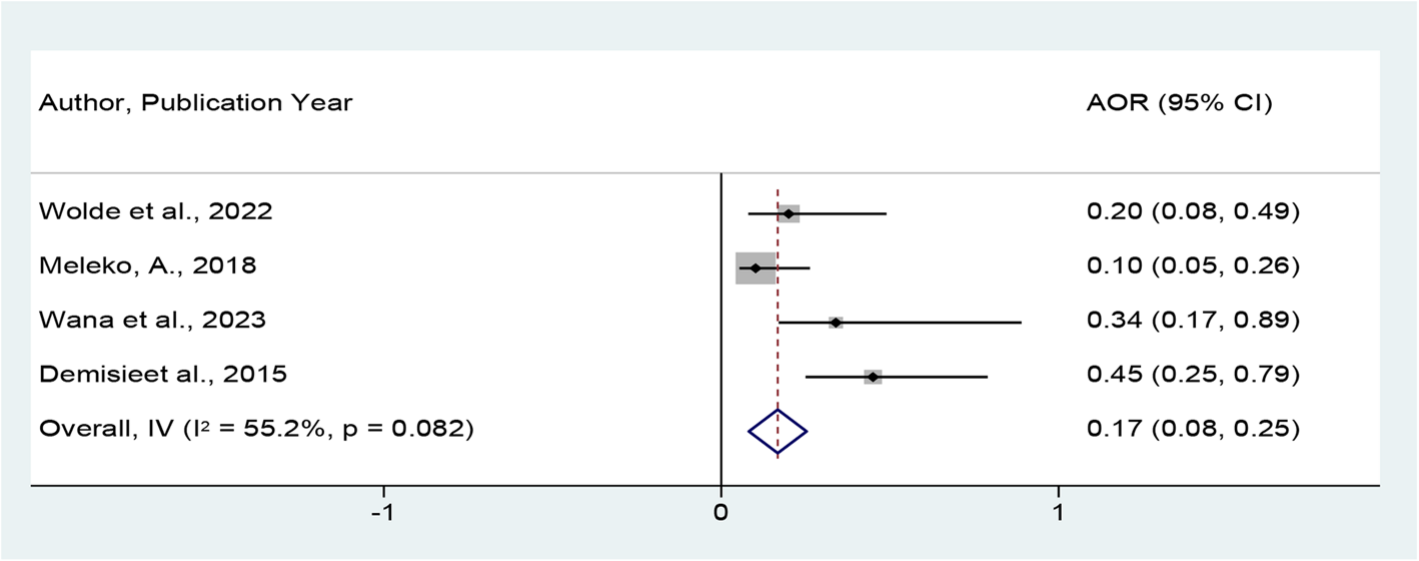
*Forest plot showing the* pooled adjusted odds ratio for the *association between* educational status *and good hand-washing at critical times in Ethiopia*

Furthermore, in this meta-analysis the pooled effects of educational level on handwashing practice at critical times were assessed by four studies [24, 30, 32, 35]. The result also indicate that mothers who had no educational level were about 83% less likely to practice good hand-washing at critical times compared to those women who had educational level (POR = 0.17, 95% CI = 0.08, 0.25) (Fig. 8).

## Discussion

This review and meta-analysis aimed to assess the pooled proportion and associated factors of good hand-washing practice at critical times among mothers of children under-five age in Ethiopia. This review found that the overall proportion of good hand-washing practice at critical times in Ethiopia was 43.18% with 95% CI (31.05, 55.31). Owing the lack of systematic reviews and meta-analyses conducted in Ethiopia, we compared the current pooled proportion with primary studies conducted broad. Thus, the result of this study are consistent with studies done in Nigeria 41.6% [14], and Pakistan 40% [36]. The findings of this study was higher than research finding reported in Uganda 28% [17] and in Indonesia 21% [15]. However, the result of this study was lower than studies conducted in Lagos, Nigeria 73.8% [37], in Bangladesh 67% [16] and in Kenya 65% [18]. This variation could be due to differences in characteristics study participant, study areas and health education and promotion program targeting the mothers on personal and environmental hygiene and lower media coverages [9, 15] and difference in the tool used for assessing good hand-washing practice [35].

A high heterogeneity was observed among the included studies. To address this, we performed a sub-group analysis by using geographical region. The overall pooled proportion of good handwashing practice at critical times was almost the same in the three geographical regions (AA, Amhara and Oromia) of Ethiopia. However, studies conducted in SNNP region of Ethiopia had the lowest proportion of proper handwashing practice at critical times. The possible justification for this difference is the awareness level of mothers. Based this findings, it may be necessary to promote the desired level of good hand hygiene at critical times in all regions of Ethiopia especially in SNNP region.

We also conducted a subgroup analysis based on the year the studies were conducted. The results indicated that studies conducted in 2019 and later reported a higher pooled prevalence of proper hand washing practices at critical times compared with those conducted before 2018.

Furthermore, a subgroup analysis was also conducted on the type of sampling methods of the included studies used. As a result, studies with simple random sampling had a higher proportion of good hand-washing practices at critical times.

Another objective of this review and meta-analysis was to identify factors that influence the proportion of good hand washing practice at critical times in Ethiopia. Accordingly, availability of water, good knowledge, desirable attitude, and higher educational status were identified as significant determinants of good hand-washing practice at critical times.

This study revealed that mothers with access to a reliable water source (taped water in home or in backyard) were more likely to practice good hand washing at critical times. This result was supported by studies conducted in rural parts of Nigeria [18, 37, 38]. This might be due to the fact that s the availability of water is the most determinant factor for good practice of hand-washing at critical times, having a reliable water source at home for proper handwashing at critical times.

This study found that mothers who had good knowledge about hand-washing was significantly associated with good handwashing practice at critical times. This study report was supported by a study results done in rural costal south India [39]and in Pakistan [36]. The possible justification might be exposure to handwashing information resulting a high level of knowledge about good hand-washing.

In addition, this meta-analysis revealed that mothers with desirable attitude about good hand-washing had a higher chance practicing good hand-washing at critical times. This was supported by previous studies conducted in Nigeria [5, 37], Uganda [17]. The possible justification could be due to the fact that information sharing and dissemination are the main determinants of behavioral change [37].

Furthermore, this meta-analysis revealed that mother’s educational status had significantly associated with good handwashing practice. Mothers who had no educational level were less likely to practice good hand-hygiene to those women who have primary and above educational level. This finding is consistent with studies conducted in in Port Harcourt, Nigeria [14], in Moradabad, India [40]. The possible reason could be those who were more educated mothers were more likely to be aware of the recommended times of hand-washing provided by health extension workers.

### Limitations of the study

Most studies included in this review and meta-analysis were sourced from one city administration and three regions of Ethiopia. The heterogeneity test indicated significant variability among the included studies, which may limit the generalisability of the findings. Due to the limited literature available on this topic, we compared our results with those of primary studies conducted abroad.

## Conclusion and recommendations

This meta-analysis revealed a pooled prevalence of 43.18% for proper handwashing practices at critical times among mothers of children below 5 years old in Ethiopia. The key factors associated with proper hand hygiene at critical times included the availability of water, adequate knowledge, educational level, and desired attitude of mothers. To promote proper handwashing practices at critical times, increasing water accessibility and providing hygiene education and training programs for improving the mothers’ knowledge and attitudes are essential.

## Data Availability

The data set of this study are available within the manuscript and its supporting information.

## Abbreviations

AA: Addis Ababa
AOR: Adjusted Odds Ratio
CI: Confidence Interval
SDGs: Sustainable development goals
SNNPR: Southern Nations Nationalities and People Region
WHO: World Health Organization
